# Feature selection algorithm based on dual correlation filters for cancer-associated somatic variants

**DOI:** 10.1186/s12859-020-03767-0

**Published:** 2020-10-30

**Authors:** Hyein Seo, Dong-Ho Cho

**Affiliations:** School of Electrical Engineering, Korea Advanced Institute of Science and Technology (KAIST), 291 Daehak-ro, Yuseong-gu, 34141 Daejeon, Republic of Korea

**Keywords:** Somatic variant, Cancer-associated variant, Feature selection, Correlation filter, Multiobjective optimization

## Abstract

**Background:**

Since the development of sequencing technology, an enormous amount of genetic information has been generated, and human cancer analysis using this information is drawing attention. As the effects of variants on human cancer become known, it is important to find cancer-associated variants among countless variants.

**Results:**

We propose a new filter-based feature selection method applicable for extracting cancer-associated somatic variants considering correlations of data. Both variants associated with the activation and deactivation of cancer’s characteristics are analyzed using dual correlation filters. The multiobjective optimization is utilized to consider two types of variants simultaneously without redundancy. To overcome high computational complexity problem, we calculate the correlation-based weight to select significant variants instead of directly searching for the optimal subset of variants. The proposed algorithm is applied to the identification of melanoma metastasis or breast cancer stage, and the classification results of the proposed method are compared with those of conventional single correlation filter-based method.

**Conclusions:**

We verified that the proposed dual correlation filter-based method can extract cancer-associated variants related to the characteristics of human cancer.

## Background

The development of next-generation sequencing (NGS), which performs high-throughput parallel sequencing of short DNA fragments, has greatly facilitated the analysis of genetic information [1]. NGS has made an important contribution to cancer research, including the understanding of cancer initiation, progression, and treatment [2–4]. Single nucleotide variant (SNV) and short insertion or deletion (InDel) are changes in genetic information of very small length and occur with very low frequencies. Variant calling algorithms for these variants, especially in the somatic cell, have been developed [5], and the close relationship between the somatic variant and the human caner has been known [6, 7].

Various methods have been applied to study the effect of somatic variant on the human cancer [8–11]. Somatic variants that could affect the selection of treatment and response of melanoma were studied based on the case-control study [8]. In [9], the driver genes for breast cancer metastasis were discovered by using the synonyms and non-synonymous ratio. A new ranking system calculating relative importance of somatic variants was proposed considering its effect size [10]. Furthermore, the pattern comparison of somatic variants between primary and metastatic tumors was performed for two colorectal cancer patients [11].

If the data to be analyzed has a large number of features, a subset of features can efficiently describe the data while reducing unrelated features [12]. Because somatic variants are high dimensional information and related to various characteristics of the individual, it is important to find variants that are closely associated with the cancer [13, 14]. In general, the feature selection method can be divided into the filter method, wrapper method, and embedded method [13, 15]. The filter method measures the importance of a subset of features according to the predefined criteria. Therefore, the filter method has less computational burdensome. On the other hand, the wrapper method is computationally expensive because it uses the prediction model with learning process to select a subset of features. Then, it usually provides very good performance. Finally, the embedded method combines the advantages of previous two methods by selecting a subset of features as part of the learning process.

Researchers have developed cancer-related feature selection algorithms for various genetic information [16–22]. In case of [16], the genetic algorithm-based feature selection method was applied to improve the decision-making process considering the tissue image of breast cancer patients. To find the set of genes for cancer classification, the quantum-behaved binary particle swarm optimization (BPSO) [17], the forward search method considering the weight local modularity [18], and the kernel-based clustering method for gene selection using double radial basis function kernels [19] were suggested. On the other hand, the identification methods of cancer-driving variants were developed by considering mutation timing of variants [20] and utilizing the gradient tree boosting and iterative search method [21]. Micro-RNA variants associated with metastasis of endometrial cancer were also analyzed using the recursive elimination technique in [22].

To understand the human cancer, it is necessary to comprehensively study the algorithm that selects the small number of variants related to the cancer’s specific characteristics. Despite previous studies, the extraction of genetic variants that are significantly associated with the cancer’s characteristics is still a difficult problem because of enormously high dimensionality of genetic information. Although the filter-based feature selection requires a relatively short time compared to other methods, the filter method also requires a lot of computations in case of genetic information. At the same time, the high performance of selection also needs to analyze complex and delicate functions of genetic information. In this paper, we propose a new modified filter-based feature selection method by improving computational complexity and classification performance for the selection of cancer-associated somatic variants. We mainly addressed the following issues here:

- The concept of dual correlation filter-based feature selection (DCFS) is proposed to extract all the significant features associated with one of the opposing characteristics while avoiding redundancy using multiobjective optimization.
- The weight value based on DCFS estimates the importance of features and is utilized to overcome the computational complexity problem of feature selection in high-dimensional data.
- The proposed DCFS-based method is applied to extract cancer-associated variants to identify melanoma metastasis or detailed stages of breast cancer. Then, the classification performance of proposed method is compared with that of conventional single correlation-based feature selection (CFS).

## Results

### Data sets

The national cancer institute (NCI) shares the genomic data of cancer through the data repository called genomic data common (GDC). We obtained annotated somatic variant files of patients with melanoma (SKCM) or breast cancer (BRCA) from the GDC portal (https://portal.gdc.cancer.gov/). Table 1 summarizes information for two data sets analysed in this paper. Regarding the melanoma metastasis, there are a total of 467 files in the SKCM set consisting of 104 primary tumors and 363 metastatic tumors. Regarding the stage II breast cancer, there are a total of 537 files for stage IIA (314) or stage IIB (223) tumors in the BRCA set. For the SKCM set, melanoma patients with primary tumors were defined as the negative class, and melanoma patients with metastatic tumors were defined as the positive class. On the other hand, for the BRCA set, we set breast cancer patients of stage IIA as the negative class and stage IIB as the positive class.

**Table 1:** Number of samples and variants of two data sets

SKCM				
Number of sample	Negative class	Positive class	All	
	Primary tumor	Metastatic tumor		
	363	104	467	
Number of variant	All	After filtering		
		Step1	Step2	Step3
	1,298,172	414,954	409,389	200,814

BRCA				
Number of sample	Negative class	Positive class	All	
	Stage IIA	Stage IIB		
	314	223	537	
Number of variant	All	After filtering		
		Step1	Step2	Step3
	424,415	88,514	75,161	37,449

Somatic variant files in SKCM and BRCA sets contain SNVs and InDels, and they were generated according to the DNA-Seq analysis pipeline. This pipeline includes the elimination of germline variants, the comparison of allele frequencies between paired normal and tumor samples, the quality control of the alignment workflow, and the annotation of each variant. VarScan2 [23] was utilized for the somatic variant calling, and FREQ value of each somatic variant was calculated. FREQ represents the proportion of reads at a particular site that contains the variant. For example, if there are 10 reads and only 6 of them have the variant at the particular location, then its FREQ value is $(6/10)\times 100 = 60\%$.

### Classification performance measurements

When there are two classes defined as the positive and negative, there are four classification results: true positive (TP), true negative (TN), false positive (FP), and false negative (FN). TP means that the data in the positive class is correctly classified as positive. On the other hand, TN means that the data in the negative class is correctly classified as negative. Conversely, FP and FN indicate that data in the positive and negative classes are miss-classified as opposite classes, respectively. The classification accuracy (*Acc*) represents the percentage of correctly categorized data as follows

1 $$\begin{aligned} Acc = \frac{TP+TN}{TP+FP+TN+FN}. \end{aligned}$$

When we consider the unbalanced data, high *Acc* can be achieved even if all data is classified into one class. In this case, $F_1$ score may be a more fair classification performance measurement. $F_1$ is the harmonic mean of the precision and recall. The precision ($Pr = \frac{TP}{TP+FP}$) calculates the number of actual positive data out of the data classified as the positive. On the other hand, the recall ($Re = \frac{TP}{TP+FN}$) calculates the number of data that are correctly classified as the positive out of all actual positive data. Then, $F_1$ is represented as follows

2 $$\begin{aligned} F_1 = \frac{2}{Pr^{-1}+Re^{-1}} = 2 \times \frac{Pr \times Re}{Pr + Re} = \frac{2 \times TP}{2 \times TP + FP + FN}. \end{aligned}$$

The range of two measurements are [0, 1]. The larger value indicates the better classification performance.

### Variant filtering results

The 3-step variants filtering was conducted for both SKCM and BRCA sets as follows.

**Step 1 **: Using ANNOVAR [24], we conducted annotations for somatic variants and identified the functional role of each variant. Then, only the variant that could directly affect protein synthesis were remained. After filtering, there were only the non-synonymous variants in coding regions.
**Step 2**: To remove somatic variants commonly detected in humans, three public databases of gnomAD, ESP6500, and ExAC were investigated, and somatic variants reported in these databases were removed. On the other hand, variants that were reported in COSMIC database [25], which is the global database of somatic variants found in human cancer, were contained in our analysis even if they were registered in gnomAD, ESP6500, or ExAC.
**Step 3**: The reliability of a somatic variant was confirmed by considering FREQ values of tumor sample and its paired normal sample. Only the variants which have FREQ $\ge 10$ in the tumor sample and have FREQ $= 0$ in its paired normal sample were extracted after filtering. On the other hand, the variants having p-value $> 0.01$ in Fisher’s t-test were removed.

As a result, we got the variants data matrix $\varvec{E}_{SKCM} \in \mathbb {R}^{467 \times 200,814}$ for SKCM set and $\varvec{E}_{BRCA} \in \mathbb {R}^{537 \times 37,449}$ for BRCA set. The number of variants for the two data sets according to the filtering steps are shown in Table 1.

### Classification using DCFS weighting algorithm with BPSO

We used the proposed DCFS weighting algorithm for cancer-associated somatic variants selection for the SKCM and BRCA sets. To confirm the performance of the proposed DCFS-based feature selection, we also applied the conventional CFS concept to the proposed weighting algorithm instead of DCFS. Pearson’s correlation coefficient (PCC), which is a basic measurement of linear correlation between two variables, was applied for correlation analysis in this study. After selecting top $D_1$ variants, BPSO [17] was applied to the selected $D_1$ variants to find the optimal set of cancer-associated variants that maximize the classification performance. The utilized parameters for the weighting algorithm and BPSO are listed in Table 2. We set $F_1$ score as the fitness function. To measure classification performance, support vector machine (SVM) [26] with k-fold cross validation was applied with $k=10$.

**Table 2:** Parameters for weighting algorithm and BPSO

Parameters for weighting algorithm	Value
Size of a random feature subset ($\Phi$)	$\{2,3, \ldots , 100\}$
Iteration time for weighting algorithm ($T_1$)	$10^6$

Parameters for BPSO	Value
Iteration time for BPSO ($T_2$)	100
Control weight (*a*)	0.5
Acceleration constants ($c_1$,$c_2$)	2
Minimum velocity ($V_{min}$)	$-6$
Maximum velocity ($V_{max}$)	6
Number of particles (*P*)	100

**Table 3:** Classification results of CFS and DCFS weighting algorithm using BPSO

$D_1$	Case	SKCM			BRCA		
		Num	$F_1$	*Acc*	Num	$F_1$	*Acc*
50	$\hbox {CFS-}D_1$	50	0.87	0.78	50	0.72	0.57
	$\hbox {CFS-}D_2$	24	0.87	0.78	23	0.74	0.58
	$\hbox {DCFS-}D_1$	50	0.87	0.77	50	0.74	0.59
	$\hbox {DCFS-}D_2$	17	0.87	0.78	25	0.74	0.59
100	$\hbox {CFS-}D_1$	100	0.87	0.77	100	0.47	0.50
	$\hbox {CFS-}D_2$	52	0.87	0.78	43	0.74	0.58
	$\hbox {DCFS-}D_1$	100	0.85	0.74	100	0.74	0.59
	$\hbox {DCFS-}D_2$	49	0.88	0.78	47	0.74	0.59
200	$\hbox {CFS-}D_1$	200	0.82	0.71	200	0.47	0.58
	$\hbox {CFS-}D_2$	88	0.87	0.78	89	0.73	0.58
	$\hbox {DCFS-}D_1$	200	0.85	0.74	200	0.73	0.58
	$\hbox {DCFS-}D_2$	87	0.88	0.79	84	0.74	0.61
300	$\hbox {CFS-}D_1$	300	0.89	0.66	300	0.42	0.56
	$\hbox {CFS-}D_2$	146	0.87	0.78	129	0.69	0.55
	$\hbox {DCFS-}D_1$	300	0.85	0.75	300	0.72	0.57
	$\hbox {DCFS-}D_2$	144	0.87	0.77	139	0.74	0.60
400	$\hbox {CFS-}D_1$	400	0.69	0.60	400	0.39	0.55
	$\hbox {CFS-}D_2$	191	0.87	0.78	200	0.48	0.58
	$\hbox {DCFS-}D_1$	400	0.85	0.74	400	0.72	0.57
	$\hbox {DCFS-}D_2$	214	0.87	0.78	199	0.74	0.60
500	$\hbox {CFS-}D_1$	500	0.64	0.56	500	0.36	0.54
	$\hbox {CFS-}D_2$	256	0.87	0.78	254	0.47	0.57
	$\hbox {DCFS-}D_1$	500	0.85	0.74	500	0.51	0.47
	$\hbox {DCFS-}D_2$	239	0.87	0.78	251	0.73	0.59

In Table 3, the number of selected variants (Num), classification accuracy (*Acc*), and $F_1$ score ($F_1$) for selected $D_1$ and $D_2$ variants are compared. $\hbox {CFS-}D_1$ and $\hbox {DCFS-}D_1$ refer to the case of using selected $D_1$ features considering CFS-weight and DCFS-weight for classification, respectively. On the other hand, $\hbox {CFS-}D_2$ and $\hbox {DCFS-}D_2$ indicate the case of using selected $D_2$ features considering CFS-weight and DCFS-weight for classification, respectively. For the SKCM set, the number of selected cancer-associated variants was reduced maintaining classification performance in the case of $\hbox {DCFS-}D_2$ than $\hbox {DCFS-}D_1$. At $D_1=400$ and $D_1=500$, the classification performance in the case of $\hbox {CFS-}D_2$ was improved compared to the case of $\hbox {CFS-}D_1$. For the BRCA set, the case of $\hbox {CFS-}D_2$ could have improved performance than the case of $\hbox {CFS-}D_1$ at $D_1 =200$ and $D_1=300$. However, classification performances at $D_1 =400$ and $D_1=500$ were not significantly improved. On the other hand, the case of $\hbox {DCFS-}D_2$ was able to choose a feature set with high classification performance in all cases.

### Classification using DCFS weighting algorithm with machine learning

Using selected $D_1$ features, we performed classifications of SKCM and BRCA sets by applying deep neural network (DNN). The utilized DNN structure consisted of two fully-connected hidden layers with 512 and 256 neurons, respectively. ReLU was used as the activation function for hidden layers, and softmax was applied to the output layer for binary classification. The batch size was 50, and the number of epochs was 100. We calculated the average *Acc* value when the $30\%$ of the randomly selected test samples were classified using the model trained with remained samples. We implemented the model using the *DNNClassifier* class from tensorflow’s *tf.estimator* module.

**Fig. 1:**
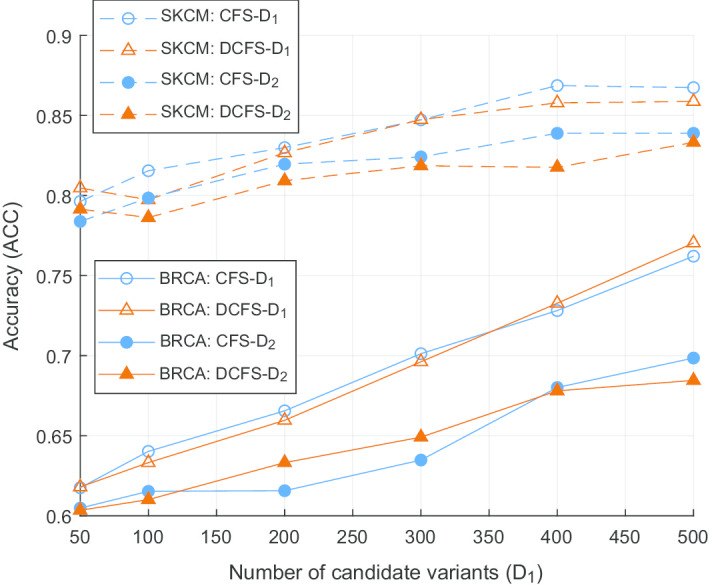
Classification results in case of using machine learning. This figure shows the classification results of two data sets using DNN classifier according to the number of selected features. For the SKCM set, DNN classifier could perform good classification in cases of using $D_1$ and $D_2$ features regardless of CFS and DCFS. For the BRCA set, DNN classifier had similar performance for CFS and DCFS methods, but the gap between the cases using $D_1$ and $D_2$ features was relatively large

Figure 1 provides the classification accuracy in the case of using $D_1$ and $D_2$ variants selected based on CFS-weight and DCFS-weight for the SKCM and BRCA sets. In general, *Acc* value of SKCM set was higher than that of BRCA set. When the number of selected features were increased, classification performances were also increased. Therefore, we could confirm that the performance of DNN classifier is affected by the number of features used for classification. Also, the classification performances of CFS-weight and DCFS-weight were similar when using the $D_1$ or $D_2$ features. For the BRCA set, the classification performance when using the $D_1$ features was relatively larger than when using the $D_2$ features. This phenomenon was also found for the SKCM set, but the gap between two cases using $D_1$ and $D_2$ features was relatively small.

## Discussion

### Pathway and phenotype analysis

To ensure the reliability of the selected significant features, variant filtering was conducted before the feature selection. However, there is no guarantee that the selected features will actually affect the phenotype of the disease [27]. Thus, in order to conclude that the features selected by the proposed method are not accidentally discovered and are actually associated with cancer, clinical studies should confirm their role in cancer biology. Several cancer-related genes are known to be associated with more than one cancer, and pathway analysis can explore biological causes by examining changes in gene expression caused by mutations. Therefore, we performed pathway and phenotype analysis for selected variants to discuss their biological significance related to human disease.

The human phenotype ontology (HPO) provides phenotypic abnormalities encountered in human disease [28]. The disease association of the gene containing the selected variant was confirmed through the HPO database. On the other hand, kyoto encyclopedia of genes and genomes (KEGG) provides a collection of pathway maps and a collection of disease entries focusing only on the perturbants [29]. We investigated HPO and KEGG databases to see if a relationship between pathway and disease was reported for the gene in which the selected variant was present. We also searched COSMIC database, which summarizes the effects of variants on human cancers.

Tables 4 and 5 provide search results for HPO, KEGG, and COSMIC databases of the selected variants in the case of using $\hbox {DCFS-}D_2$ at $D_1=50$ for the SKCM and BRCA data sets, respectively. For the SKCM set, 5 genes and 6 genes were found in HPO and KEGG databases, and 6 variants were reported in COSMIC database. Among the 17 selected variants, there were 3 variants that were not registered in any of the three databases. For the BRCA set, information about 6 genes and 10 genes were collected from HPO and KEGG databases, respectively. In the case of COSMIC database, 18 variants among 25 selected variants were reported in association with human cancer. On the other hand, there were 3 variants that were not detected in three databases.

**Table 4:** Selected variants for SKCM in case of using $\hbox {DCFS-}D_2$ at $D_1 = 50$

CHR	START	REF	ALT	GENE	HPO	KEGG	COSMIC
1	190098828	C	T	BRINP3			COSM1689444
2	37231652	G	A	NDUFAF7		ko04714	
2	137450953	G	A	THSD7B			
2	178756687	C	T	TTN	OMIM:604145; OMIM:608807; ORPHA:169186; OMIM:600334; OMIM:611705; OMIM:613765; OMIM:603689; ORPHA:324604; ORPHA:609	ko05410; ko05414; H00292; H00294; H00593; H00594; H01976	COSM1482258; COSM1482259; COSM1482261; COSM1482257; COSM1482260
4	137531580	G	A	PCDH18			COSM3428175
5	13753490	G	A	DNAH5	ORPHA:244; OMIM:608644		COSM1695413
6	56067320	C	T	COL21A1			COSM1445258; COSM1445259
8	76852088	C	T	ZFHX4	OMIM:178300		
9	127937878	C	T	DPM2	ORPHA:329178; OMIM:615042	ko00510; ko00563; ko01100; H00118	
11	55367976	G	A	OR4A15			COSM106310
11	61792641	G	A	FEN1		ko03030; ko03410; ko03450	
15	64163030	G	A	PPIB	OMIM:259440	H00506	
16	20032148	G	A	GPR139			COSM967969
19	45691963	C	T	SNRPD2		ko03040	
19	45691983	C	T	SNRPD2		ko03040	
20	35542046	G	A	ERGIC3			
20	42098471	C	T	PTPRT

**Table 5:** Selected variants for BRCA in case of using $\hbox {DCFS-}D_2$ at $D_1 = 50$

CHR	START	REF	ALT	GENE	HPO	KEGG	COSMIC
1	43305332	G	T	TIE1			COSM3805284; COSM3805283
2	46576099	C	G	RHOQ		ko04910	
2	131528196	G	C	CCDC74A			COSM1752030
2	178562952	G	C	TTN	OMIM:604145; OMIM:608807; ORPHA:169186; OMIM:600334; OMIM:611705; OMIM613765; OMIM:603689; ORPHA:324604; ORPHA:609	ko05410; ko05414; H00292; H00294; H00593; H00594; H01976	COSM1482258; COSM1482259; COSM1482261; COSM1482257; COSM1482260
3	37347240	G	A	GOLGA4			COSM1044027
5	31401487	C	A	LVRN			
5	36152895	G	T	SKP2		ko04068; ko04110; ko04120; ko04150; ko05169; ko05200; ko05203; ko05222	
5	115983289	C	G	DROSHA		ko03008; ko05205	COSM3827928
6	47682558	G	T	ADGRF2			
6	83168072	A	C	DOPEY1			COSM3831123; COSM3831122
8	19405982	C	T	CSGALNACT1		ko00532; ko01100	COSM454271
10	50841872	A	T	A1CF			COSM3807316; COSM3807318; COSM3807317
11	78812245	T	C	TENM4	OMIM:616736		
14	19920797	G	A	OR4K5			COSM1663253
14	23386104	C	A	MYH6	OMIM:613251; OMIM:613252; OMIM:614089; OMIM:192600; ORPHA:154	ko04022; ko04260; ko04261 ko04919; ko05410; ko05414; ko05416; H00292; H00294; H00546; H00594; H00656; H00703; H01216; H01977	COSM1477478
14	67725248	C	A	ZC3H14	ORPHA:88616; OMIM:617125	H00768	COSM1477814
14	76831292	C	T	RDH12	ORPHA:791; ORPHA:65; OMIM:612712	ko00830; ko01100; H00837	COSM3815158
14	88572068	G	C	LRRC74A			COSM3815383
15	48168740	C	A	MYEF2			COSM1373221
17	37274266	G	A	ACACA	OMIM:613933	ko00061; ko00254; ko00620;ko00640; ko01100; ko01110; ko01120; ko01130; ko01212; ko04152; ko04910; ko04922	
19	22304762	G	A	ZNF729			COSM439106
19	37635574	C	T	ZFP30			
19	39390264	C	A	ZNF575			COSM3823309
19	43534433	G	A	PAF1		ko04011	COSM3823010
20	44614698	G	A	PKIG			COSM443871

*BRAF*, *NRAS*, and *KIT* are three well-known genes associated with melanoma, and *BRCA1* and *BRCA2* are two representative genes associated with breast cancer. The genes that play a role in inducing or inhibiting metastasis have been actively studied, but have not yet been clearly identified. Also, the genes involved in cancer progression are well known, but the detailed progression of subgroup classification of stage II breast cancer has not been addressed. Since we confirmed the relationship between cancer and disease for most of the extracted genes, it is worth to study the biological function of the extracted genes for melanoma metastasis or subgroup of stage II breast cancer through clinical studies.

### Effect of correlation analysis method

To compare the impact of correlation analysis method on the proposed DCFS method, we considered PCC and Spaerman’s rank correlation coefficient (SCC). PCC is a famous measure of the correlation between two data sets. PCC is defined as

3 $$\begin{aligned} \rho _{P}(\varvec{a},\varvec{b}) = \frac{\sum _i (a_i-\mu _{\varvec{a}})(b_i-\mu _{\varvec{b}})}{\sqrt{\sum _i (a_i-\mu _{\varvec{a}})^2} \sqrt{\sum _i (b_i-\mu _{\varvec{b}})^2}} \end{aligned}$$

where $\mu _{\varvec{a}}$ and $\mu _{\varvec{b}}$ refer to the mean value of the vector $\varvec{a}$ and $\varvec{b}$, respectively. The range of PCC values is $[-1, 1]$. The closer to 1, the higher is the positive correlation. Conversely, the closer to -1, the higher is the negative correlation, and 0 means no correlation. PCC measures linear relationships between two vectors $\varvec{a}$ and $\varvec{b}$. On the other hand, SCC can consider nonlinear relationships where the amount of change is not constant. Instead of raw data $\varvec{a}$ and $\varvec{b}$, SCC is calculated based on ranking values $r\varvec{a}$ and $r\varvec{b}$:

4 $$\begin{aligned} \rho _{S}(\varvec{a},\varvec{b}) = \rho _{P}(r\varvec{a},r\varvec{b}) = \frac{\sum _i (ra_i-\mu _{r\varvec{a}})(rb_i-\mu _{r\varvec{b}})}{\sqrt{\sum _i (ra_i-\mu _{r\varvec{a}})^2} \sqrt{\sum _i (rb_i-\mu _{r\varvec{b}})^2}} \end{aligned}$$

where $\mu _{r\varvec{a}}$ and $\mu _{r\varvec{b}}$ refer to the mean value of $r\varvec{a}$ and $r\varvec{b}$, respectively. The range of SCC values is also $[-1, 1]$.

**Fig. 2:**
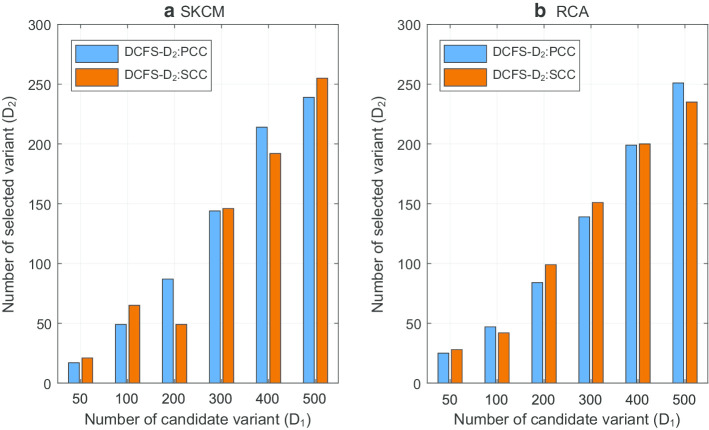
Impact of correlation analysis method on number of selected features. In this figure, the number of selected features when PCC and SCC are utilized for the proposed DCFS weighting algorithm with BPSO are compared. The number of selected features depended on the data set and $D_1$, and the influence of PCC and SCC was small

**Fig. 3:**
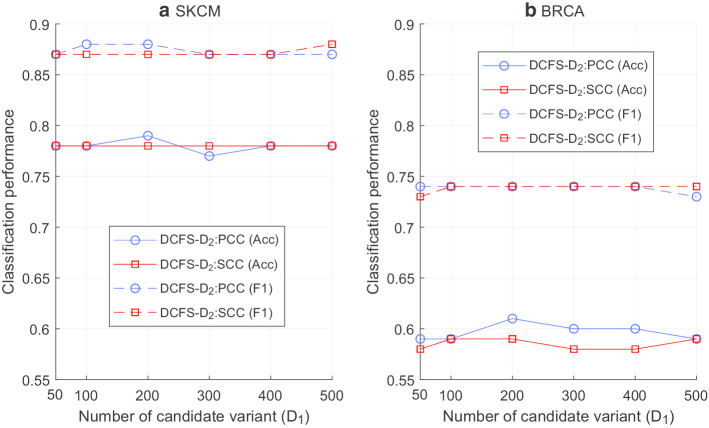
Impact of correlation analysis method on classification performance. This figure compares classification results of two data sets when PCC and SCC are utilized for the proposed DCFS weighting algorithm with BPSO. The classification performance was similar when using the two correlation analysis methods for both SKCM and BRCA sets

Based on PCC and SCC, DCFS-weighting algorithm selected $D_1$ variants, and BPSO was applied to get $\hbox {DCFS-}D_2$. Figure 2 illustrates the number of selected variants according to $D_1$. In general, PCC and SCC selected similar number of features for both SKCM and BRCA data sets. Furthermore, classifications were performed using SVM and k-fold cross validation with $k=10$ for the SKCM and BRCA data sets. Figure 3 compares the classification performances when PCC and SCC are utilized for $\hbox {DCFS-}D_2$ according to $D_1$, respectively. In case of the number of selected variants, it was hard to see any special trends according to the selection of correlation analysis method. Also, the classification performances were similar for PCC and SCC. As a result, the choice of correlation analysis method had little effect on the classification performance of SKCM and BRCA data sets. When the correlation between two variables is week, the difference between the PCC and SCC values is small. However, when the correlation is strong, the difference between the two values becomes larger depending on whether the correlation is linear. In the SKCM and BRCA data sets, there were very few variants with strong correlations, and the impact of correlation analysis method seems to be small.

### Complexity

CFS is a filter-based feature selection method and has the advantage of very fast computation. The complexity of the CFS merit maximization of Eq. (5) depends on the use of optimization methods. The proposed DCFS merit just needs a modified CFS merit calculation of Eq. (6). Therefore, the increase of computational complexity of DCFS compared to CFS is insignificant. When there are *V* variants and *S* samples with $V>> S$, the calculation complexity of DCFS merit follows $O(V^2)$, which is the same as the conventional CFS merit calculation.

To find the optimal subset of features maximizing the DCFS merit, $2^V-1$ calculations of the DCFS merit are required, and the complexity becomes $O(2^VV^2)$. The proposed DCFS weighting algorithm can reduce the number of the DCFS merit calculation whose complexity is denoted as $O(T_1V^2)$ with $T_1 \le 2^V$.

## Conclusions

In this study, we proposed the concept of DCFS to analyze cancer-associated somatic variants, containing SNVs and InDels. In order to reduce the computational complexity and to eliminate the effects of errors and biases, non-significant variants were removed considering the functional role, previous studies of disease association, and the reliability of variant. The DCFS merit was defined based on the multiobjective optimization to obtain the cancer-associated variants set related to activation and deactivation of the cancer’s characteristics without redundancy. Because of high dimensionality of genetic information, we suggested DCFS weighting algorithm to reduce the complexity of feature selection procedure. We applied our proposed algorithm to identify metastasis of melanoma or the subgroup of stage II breast cancer. BPSO was used for DCFS maximization for significant variant selection, and a neural network was applied for classification of data. In addition, pathway and phenotype analysis were performed to study the effects of the variants selected by the proposed algorithm on the cancer phenotype. As a result, we verified that proposed DCFS algorithm could select cancer-associated variants resulting in high classification performance. We also discussed the impact of the choice of correlation analysis method on the proposed method. In summary, we believe that the proposed method can be applied to various analysis of genomic data and various feature selection analysis.

## Methods

### Variant filtering

**Fig. 4:**
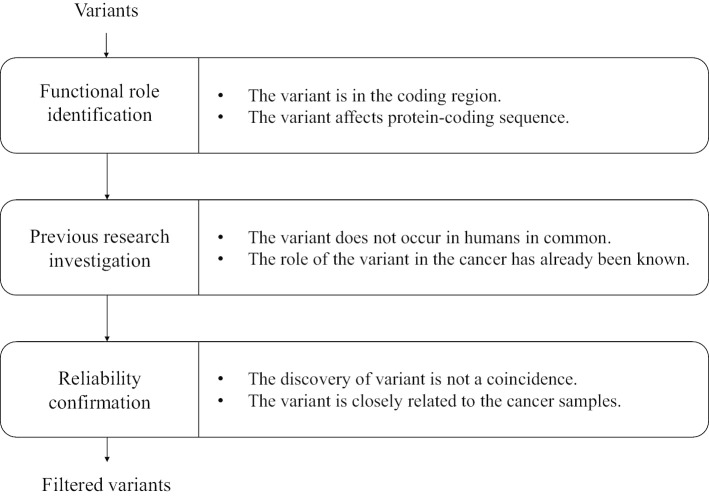
3-step variant filtering procedure. Three steps of filtering for cancer-associated variants extraction are illustrated in this figure. In the first step, we identified the functional role of variant considering whether it directly affects protein synthesis. Next, we searched public databases to eliminate non-critical variants, and also referred to previous studies to include variants known to be important for the cancer. Finally, the last step confirmed that the variant did not occur accidentally during the sequencing or variant calling and was deeply related to the cancer samples

Somatic variants, containing SNV and InDel, can be detected from NGS data by using various variant calling methods such as VarScan2 [23] and SomaticSniper [30]. After the variant calling, there are too many variants, and the non-critical variants also can be contained. We can remove the non-critical variants considering the functional role of variant, previous researches on variant, and the reliability of variant. The 3-step filtering procedure is summarized in Fig. 4.

**Step 1 **: The functional role of variant is identified. The variants in non-coding regions that affect cancer have been studied [31, 32]. However, the analysis of variant in the non-coding region is still more challenging than in the coding region, because it is difficult to interpret the functional role of variant. We focus on studying variants in coding regions that are directly related to protein synthesis. Therefore, only the variants in coding regions including exons, 3$'$ UTR, and 5$'$ UTR are extracted. Even if the variant is in the coding region, the amino acid sequence may not be modified and may not cause actual protein-coding change. This silent variation is called synonymous variant and removed with ambiguous variants, which are caused by error in sequencing and calling procedures.
**Step 2**: The previous researches on variant are investigated. The significant variant related to cancer is not commonly detected in humans. Therefore, we exclude the variant if it is already registered in public databases. However, the variant is re-included in the study if previous studies have reported the effect of the variant in the human cancer.
**Step 3**: The reliability of variant is confirmed. For the quality control of data, it should be confirmed that the variant dose not occur accidentally during the process of variant acquisition. Also, it is necessary to take into account the association with the cancer. Therefore, the variant is selected when the reads having the variant occur frequently in cancer samples and do not occur in normal samples.

### DCFS

There are two types of cancer-associated variants that encourage and suppress the expression of certain characteristic of cancer. Both types of cancer-associated variants should be considered. However, if we extract these two types of variants separately, some of extracted variants can be included in both types. Therefore, we propose the concept of DCFS utilizing the multiobjective optimization to eliminate these redundant variants and generalize the correlation-based cancer-associated variants selection.

In [33], a filter-based feature selection method based on the correlation of data called CFS was proposed. CFS approach selects the least number of features that are closely related to the data class. In other words, CFS selects a set of features that are strongly correlated with the class but not each other. The merit criterion of CFS for a feature set $\varvec{f}$ consisting of *n* features are as follows:

5 $$\begin{aligned} M_{CFS}=\frac{n \overline{r_{\varvec{f}\varvec{c}}}}{\sqrt{n + n (n-1) \overline{r_{\varvec{f}\varvec{f}}}}}, \end{aligned}$$

where $\overline{r_{\varvec{f}\varvec{c}}}$ is the mean of the correlations between a feature and the class, and $\overline{r_{\varvec{f}\varvec{f}}}$ is the mean of the correlations between two features. The optimal subset of features with the maximum merit is selected.

The proposed DCFS extends CFS to find the smallest feature subset associated with two conflicting classes of data. The set of significant features in DCFS satisfies the following two conditions:

- The selected feature is highly correlated with only one class.
- The selected features are not correlated with each other.

The first condition constrains the selected feature to be not correlated to both opposing characteristics. The second condition encourages that there is no duplicate information in the selected feature set. Let the data can be divided as two classes: positive or negative. Then, we can define two merit criterion $M_p$ and $M_n$. In the case of $M_p$, the selected features are the set of significant features specifically associated with the positive class. Also, in the case of $M_n$, the selected features are specifically related to the negative class. DCFS maximizes $M_p$ and $M_n$ taking into account the relationship between features and the two classes simultaneously.

Let the data matrix be $\varvec{E}$, where an element $e_{sv}$ refers *v*-th feature of *s*-th sample for all $v \in \{1, 2, \ldots ,V\}$ and $s \in \{1, 2, \ldots , S\}$. Then, a column vector $\varvec{e}_{v}$ means values of *v*-th feature of all *S* samples. Also, the column vector $\varvec{c}^p$ and $\varvec{c}^n$ represent the positive and negative class index of samples, respectively. If the selection vector is $\varvec{x}$, where an element $x_v \in \{0, 1\}$ for all $v \in \{1, 2, ...,V\}$, $x_v = 1$ means that *v*-th feature is selected. On the other hand, $x_v = 0$ means *v*-th feature is not selected. To consider both objective functions $M_p$ and $M_n$ at the same time, we use the multiobjective optimization problem. Then, $\varvec{x}$ is determined by following equation:

6 $$\begin{aligned}&\mathop {\hbox {argmax}}\limits _{\varvec{x}} \; M_{DCFS} \\&\quad = \mathop {\hbox {argmax}}\limits _{\varvec{x}} \; \alpha M_{p} + (1-\alpha )M_{n} \\&\quad = \mathop {\hbox {argmax}}\limits _{\varvec{x}} \; \alpha \frac{|\varvec{x}| {\overline{r_{\varvec{e}\varvec{c}^p}(\varvec{x})}}}{\sqrt{|\varvec{x}| + |\varvec{x}| (|\varvec{x}|-1) {\overline{r_{\varvec{ee}}(\varvec{x})}}}} + (1-\alpha )\frac{|\varvec{x}| {\overline{r_{\varvec{e}\varvec{c}^n}(\varvec{x})}}}{\sqrt{|\varvec{x}| + |\varvec{x}| (|\varvec{x}|-1) {\overline{r_{\varvec{ee}}(\varvec{x})}}}}. \end{aligned}$$

where $\alpha \in [0,1]$ is a scalarization parameter, $|\varvec{x}| = \sum _v {x_v}$ is the number of selected features, $\overline{r_{\varvec{ee}}(\varvec{x})}$ is the mean of the correlations between any two features, $\overline{r_{\varvec{e}\varvec{c}^p}(\varvec{x})}$ is the mean of the correlations between a feature and the class index $\varvec{c}^p$, and $\overline{r_{\varvec{e}\varvec{c}^n}(\varvec{x})}$ is the mean of the correlations between a feauture and the class index $\varvec{c}^n$. These three mean correlation values are defined as

7 $$\begin{aligned} \overline{r_{\varvec{ee}}(\varvec{x})}= & {} \frac{1}{|\varvec{x}| (|\varvec{x}|-1)} \sum _{\forall i,j \ne i} \rho (\varvec{x} \cdot \varvec{e}_{i}, \varvec{x} \cdot \varvec{e}_{j}) \end{aligned}$$

8 $$\begin{aligned} \overline{r_{\varvec{e}\varvec{c}^p}(\varvec{x})}= & {} \frac{1}{|\varvec{x}|} \sum _{i} \rho (\varvec{x} \cdot \varvec{e}_{i},\varvec{c}^p) \\ \overline{r_{\varvec{e}\varvec{c}^n}(\varvec{x})}= & {} \frac{1}{|\varvec{x}|} \sum _{i} \rho (\varvec{x} \cdot \varvec{e}_{i},\varvec{c}^n) \end{aligned}$$

where $\varvec{a} \cdot \varvec{b}$ refers the element-wise multiplication between the vectors $\varvec{a}$ and $\varvec{b}$ of the same length, and $\rho (\varvec{a},\varvec{b})$ is the correlation coefficient between $\varvec{a}$ and $\varvec{b}$. In Eq. (6), the scalarization parameter $\alpha \in [0,1]$ adjusts the importance of the two objective functions, which are $M_p$ and $M_n$. For the multiobjective optimization problem, there may not be a single solution because multiple objective functions can conflict with each other. In this case, there is one or more Pareto optimal solutions. Pareto solutions mean that we need to reduce other objective values to improve one objective value. The linear scalarization finds the most appropriate Pareto optimal solution using the parameter $\alpha$. When $\alpha = 1$, the correlation with the positive class determines the objective function and only the positive class related features are extracted. Conversely, if $\alpha = 0$, the features associated with the negative class are selected considering the correlation with the negative class. If let $\alpha = 0.5$, the problem fairly considers two objective functions, and significant features related to both classes are extracted without duplicating information.

### DCFS weighting algorithm

If the data dimension is very large, selecting the optimal subset of features based on the filter method is also complex. Therefore, we define the DCFS-weight to indicate the expected importance of each feature. Then, we can pre-select candidate critical features to alleviate the computational complexity problem.

To calculate the DCFS-weight for each feature, the proposed DCFS weighting algorithm iteratively performs DCFS calculation on a randomly selected subset of features. Let the number of iteration be $T_1$. The sum of DCFS values of each feature is defined as the vector $\varvec{w}^{val} = ({w}^{val}_1, {w}^{val}_2, \ldots , {w}^{val}_V)$. Similarly, $\varvec{w}^{num} = ({w}^{num}_1, {w}^{num}_2, \ldots , {w}^{num}_V)$ is defined to count the number of times that each feature is selected during $T_1$ iterations. Both $\varvec{w}^{val}$ and $\varvec{w}^{num}$ are initialized with a zero vector and updated through $T_1$ iterations. At a *t*-th iteration, a size of feature subset $\phi ^t \in \Phi$ is randomly determined, and $\phi ^t$ features are randomly selected among *V* features. Then, DCFS value, which is $M_{DCFS}(\varvec{E}^t)$, is calculated from $\varvec{E}^t$, which is the data matrix only for selected feature subset $\varvec{I}^t$, and ${w}^{val}_i$ and ${w}^{num}_i$ for all $i \in \varvec{I}^t$ are updated. $M_{DCFS}(\varvec{E}^t)$ is added to ${w}^{val}_i$ for all $i \in \varvec{I}^t$, and ${w}^{num}_i$ is increased by 1 for all $i \in \varvec{I}^t$. After $T_1$ iterations, we can calculate the DCFS-weight vector as follows

9 $$\begin{aligned} \varvec{w} = ({w}_1, {w}_2, \ldots , {w}_V) = \left( \frac{{w}^{val}_1}{{w}^{num}_1}, \frac{{w}^{val}_2}{{w}^{num}_2}, \ldots , \frac{{w}^{val}_V}{{w}^{num}_V}\right) . \end{aligned}$$

By using the DCFS weighting algorithm, we can calculate the DCFS-weight $\varvec{w}$, and top $D_1$ features are extracted as the candidate significant features.

After using the DCFS weighting algorithm, we can get the data matrix $\varvec{E}'$ only for the selected $D_1$ features. By considering $D_1<< V$ features, the DCFS merit optimization based on Eq. (6) requires lower computational complexity compared to the case considering all *V* features. BPSO [17] is applied to find the optimal set of features by maximizing $M_{DCFS}(\varvec{E}')$. Particle swarm optimization (PSO) is an optimization method inspired by the social behavior of bird or fish groups [34, 35]. BPSO was developed for the discrete search space by modifying PSO and can be applied to the feature selection [17, 36]. A feature selection is defined as a particle and is represented by a position vector that indicates whether each feature is selected or not. Each particle moves in the search space with the dimension $D_1$ and updates its position information repeatedly to maximize the fitness function. The position vector of a particle at *t*-th iteration is represented as $\varvec{x}_p^t = (x_{p1}^t,x_{p2}^t, \ldots , x_{pD_1}^t)$, and its movement velocity is $\varvec{v}_p^t = (v_{p1}^t,v_{p2}^t, \ldots , v_{pD_1}^t)$ for all paticles $p \in \{1, 2, \ldots , P\}$. At the $(t+1)$-th iteration, the velocity and position vectors are updated as follows

10 $$\begin{aligned} v_{{pd}}^{{t + 1}} & = av_{{pd}}^{t} + c_{1} r_{1} (pb_{{pd}}^{t} - x_{{pd}}^{t} ) + c_{2} r_{2} (gb_{d}^{t} - x_{{pd}}^{t} ) \\ x_{{pd}}^{{t + 1}} & = \left\{ {\begin{array}{*{20}l} 1 \hfill & {{\text{if}}\;rand} \hfill & { < \frac{1}{{1 + e^{{ - v_{{pd}}^{{t + 1}} }} }}} \hfill \\ 0 \hfill & {{\text{otherwise}}{\text{.}}} \hfill & {} \hfill \\ \end{array} } \right.{\text{ }} \\ \end{aligned}$$

where *a* is a weight value that controls the effect of the previous velocity, $c_1$ and $c_2$ are acceleration constants, $r_1$ and $r_2$ are random values in [0, 1] that follow the uniform distribution. Also, $\varvec{pb}_p^{t}=({pb}_{p1}^{t},{pb}_{p2}^{t}, \ldots , {pb}_{pD_1}^{t})$ refers the best position of particle *p* known during *t* iterations, and $\varvec{gb}^{t}=({gb}_{1}^{t},{gb}_{2}^{t}, \ldots , {gb}_{D_1}^{t})$ refers the global best position among all the particles found during *t* iterations. The range of $v_{pd}^{t+1}$ is restricted to $[V_{min},V_{max}]$. For the feature selection, the element of position vector is restricted to $\{0, 1\}$ using the sigmoid function. After updating $\varvec{x}_p^{t+1}$ and $\varvec{v}_p^{t+1}$ for all $p \in \{1, 2, \ldots , P\}$, the fitness function $M_{DCFS}(\varvec{E}')$ for all particles are calculated, and $\varvec{pb}_p^{t+1}$ and $\varvec{gb}^{t+1}$ are defined for the next iteration. After $T_2$ iterations, the global best position $\varvec{gb}^{T_2}$ indicate the optimal feature subset consisting of $D_2 = \sum _{d=1}^{D_1} {gb}_{d}^{T_2}$ features. Then, the features with ${gb}_{d}^{T_2} = 1$ for all $d \in \{1,2, \ldots , D_1\}$ are selected. The pseudo-code of the proposed DCFS-based feature selection containing the DCFS weighting algorithm is shown in Algorithm 1.
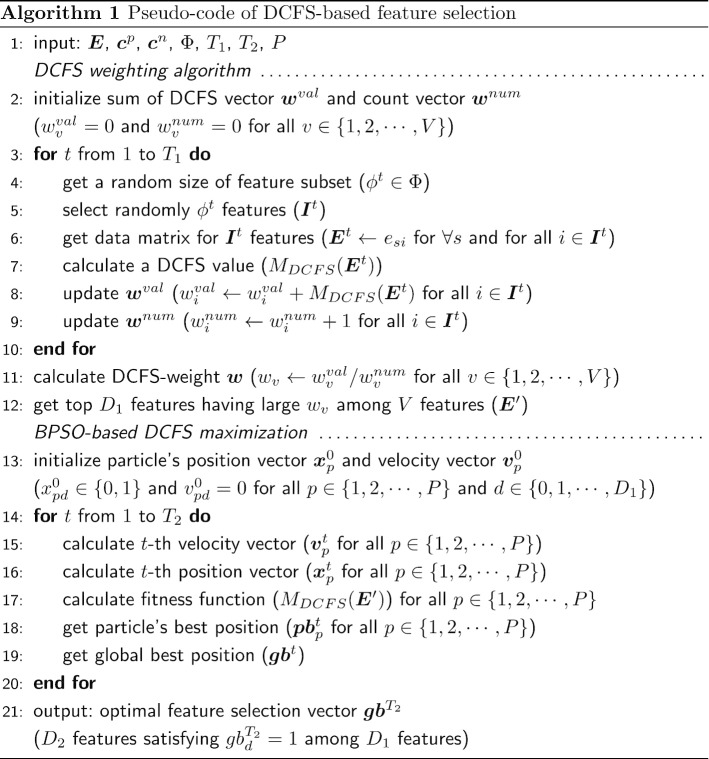


## Data Availability

The datasets analysed during the current study are available in the NCI GDC repository, https://portal.gdc.cancer.gov/.
